# The Effect of Withdrawal Rate on Crystal Structure Perfection, Microstructure and Creep Resistance of Single Crystal Castings Made of CMSX-4 Nickel-Based Superalloy

**DOI:** 10.3390/ma12203422

**Published:** 2019-10-19

**Authors:** Kamil Gancarczyk, Maciej Zubko, Aneta Hanc-Kuczkowska, Barbara Kościelniak, Robert Albrecht, Dariusz Szeliga, Maciej Motyka, Waldemar Ziaja, Jan Sieniawski

**Affiliations:** 1 Department of Materials Science, The Faculty of Mechanical Engineering and Aeronautics, Rzeszow University of Technology, 12 Powstancow Warszawy Ave., 35-959 Rzeszow, Poland; b.koscielnia@prz.edu.pl (B.K.); dszeliga@prz.edu.pl (D.S.); motyka@prz.edu.pl (M.M.); wziaja@prz.edu.pl (W.Z.); jansien@prz.edu.pl (J.S.); 2 Research and Development Laboratory for Aerospace Materials, Rzeszow University of Technology, 4 Zwirki i Wigury Str., 35-036 Rzeszow, Poland; 3 Institute of Materials Science, University of Silesia, 1a 75 Pulku Piechoty Str., 41-500 Chorzow, Poland; maciej.zubko@us.edu.pl (M.Z.); aneta.hanc@us.edu.pl (A.H.-K.); robert.albrecht@us.edu.pl (R.A.); 4 Department of Physics, University of Hradec Kralove, Rokitanshego 62/26 Str., 500-03 Hradec Kralove, Czech Republic

**Keywords:** nickel-based superalloy, CMSX-4, single crystal (SX), crystal structure perfection, creep resistance

## Abstract

This study focuses on the evaluation of the crystal structure perfection in the single crystal made of CMSX-4 nickel superalloy and its effect on creep resistance. Single crystal castings were manufactured by directional solidification process at the withdrawal rate of 1, 3, 5 and 7 mm/min. Light (LM) and electron (SEM, TEM) microscopy, X-ray diffraction and Mossbauer spectroscopy were used for evaluation of the microstructure and crystal structure perfection. Castings were also subjected to creep tests. The best creep resistance was obtained for the casting manufactured at the withdrawal rate of 3 mm/min, characterized by the highest crystal structure perfection compared to the other castings examined.

## 1. Introduction

Single crystal (SX) components (blades) of the hot section in aircraft engines are manufactured by directional solidification process. They are submitted to quality control, including the assessment of crystal orientation. The deviation angles between relevant crystallographic directions—required (for perfect crystal) and real—in produced SX casting are compared. These values are considered as main factors for evaluation of the degree of crystal structure perfection. The structure of SX casting is formed during solidification process of a single nucleus along one crystallographic direction. In most cases, it is the [001] direction, in order to provide high creep resistance. Therefore, creep behavior of a single crystal is determined by its structure perfection [1,2,3,4,5,6].

The technological process based on the Bridgman–Stockbarger method is the most commonly used in industry for manufacturing nickel-based superalloy single-crystal blades. It is composed of several stages: development of a three-dimensional 3D model of casting, preparation of a wax model, making a ceramic form, wax removal and annealing of the ceramic form, pouring liquid metal into the mold and conducting directional solidification. The process of directional solidification of nickel-based superalloy single crystals under the Bridgman (or Bridgman–Stockbarger) method is usually carried out in a vacuum furnace of vertical construction—the ceramic mold is slowly withdrawn from the heating zone [7,8,9,10,11].

**Figure 1:**
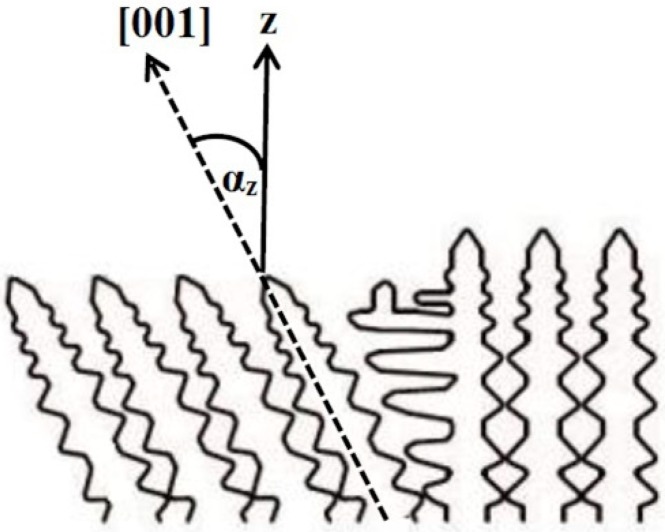
Angle α_z_ between crystallization and crystal [001] directions in a scheme of the dendritic structure.

A nickel-based CMSX-4 superalloy is used for fabrication the SX blades of high-pressure turbines working in extreme conditions in aircraft jet engines. The exceptional properties of the CMSX-4 superalloy are obtained due to its microstructure composed of two basic components—γ and γ’ phase crystals (Figure 1). The matrix is a solid solution, disordered γ phase having cubic structure (cF4) with space group (Fm3m) and lattice parameter a_0γ_ = 0.352 nm. The intermetallic strengthening γ’ phase Ni3Al(Ti,Ta) is partly ordered and characterized by a cubic crystal structure (cP4) with space group (Pm3m) and lattice parameter a_0γ’_ = 0.356 nm [12,13,14,15,16,17].

Alloying elements of CMSX-4 superalloy can be classified in terms of their influence on the microstructure and properties:

- Strengthening of γ solid solution–Co, Cr, Mo, Re, Ru and W,
- Forming precipitations of the strengthening phase γ’-Al, Ta and Ti.

The solidification process leads to heterogeneity of both chemical composition and microstructure. Increase of the withdrawal rate in the Bridgman process favors the segregation of alloying elements—Al, Ta, Re, Ti and W—and leads to heterogeneous precipitation of the strengthening γ’ phase. The analysis of the literature data indicates that the withdrawal rate vw of SX blades under industrial conditions takes a value in the range from 1 to 5 mm/min [18,19,20].

Creep resistance of SX turbine blades, especially used for the first stage of the turbine, can be improved by elimination of high-angle grain boundaries. Such structural parts are manufactured in the investment casting process carried out in a vacuum condition using multi-layered ceramic molds. It is of a great importance that solidification parameters—pouring temperature and withdrawal rate—of mold are precisely controlled and modified as needed. In order to achieve high quality of the products, continuous development of materials and components used in the technological process, such as wax models, ceramic molds and others, is required [21,22].

Moreover, another important factor that determines the creep resistance of a turbine blade is the single crystal orientation. The high creep strength of SX turbine blades requires that the direction of crystal withdrawal is parallel or only slightly deviated from the [001] crystallographic direction (deviation angle α_z_) [23]. The high deviation of the single crystal leads to a decrease in the mechanical properties of superalloys. Therefore, it is assumed that the value of the deviation angle αz should be lower than 15° [24].

The conditions of crystallization determine the occurrence of porosity and shrinkage in castings. Hence, the pore size is directly related to the crystallization conditions and microstructural parameters. It was found that the pore size increased with increasing withdrawal rate of the single crystal castings. Porosity determines the mechanical properties of SX castings made of nickel-based superalloy and it is an important evaluation criterion of their quality [25,26,27,28,29,30].

In this work, an assessment of the influence of crystallization process conditions—mainly the withdrawal rate—on the microstructure and crystal orientation of the SX casting was made. Test methods such as light microscopy, scanning and transmission electron microscopy, X-ray diffraction and Mossbauer spectroscopy were used for material characterization. The combination of these methods allowed for an accurate assessment of the microstructure of single crystals. Creep tests were also carried out to analyze the mechanical strength of produced castings. Obtained data can be useful for the selection of crystallization process conditions for nickel-based SX castings.

## 2. Experimental

Single crystal rods in the as-cast condition made of commercial CMSX-4 nickel-based superalloy (Table 1) were studied. The lost-wax casting method, including multilayer ceramic shell preparation, was used to obtain single crystal casting. The directional solidification process (based on the Bridgman method) was carried out in a vacuum furnace ALD VIM-IC 2 E/DS/SC (Hanau, Germany) at various withdrawal rates of v_w_ = 1, 3, 5 and 7 mm/min. Before pouring, the mold had been heated up to the temperature of 1500 °C, for 2 h. The temperature of liquid metal poured into the mold was 1550 °C. The entire manufacturing process was carried out in the Department of Materials Science and Research and Development Laboratory for Aerospace Materials at Rzeszow University of Technology.

Microstructure evaluation of the CMSX-4 superalloy in the as-cast condition was performed using light microscope (LM) Leica DMI3000M (Wetzlar, Germany) and scanning electron microscope (SEM) Hitachi S-3400N (Tokyo, Japan). Samples for LM and SEM observation were prepared by chemical etching in a solution containing 3 g MoO_3_, 100 mL HCl, 100 mL HNO_3_ and 100 mL H_2_O. The distance between primary dendrite arm spacing (PDAS) was measured for five images of the microstructure of one sample to determine its mean value.

The quantitative analysis of the microstructure was performed to determine the following: PDAS, surface area S_γ’_ and volume fraction V_vγ’_ of γ’ phase precipitates and number of the pores. Several LM and SEM microstructure images for each withdrawal rate were processed by the Leica Application Suite v3.7 software in accordance with procedures described in the literature [31]. The chemical composition of γ’ phase was investigated by transmission electron microscope (TEM) JEOL JEM-3010 (Tokyo, Japan) equipped with EDS detector (Energy Dispersive X-ray Spectroscopy, Thermo Scientific, Waltham, MA, USA). For determining the lattice parameter of the γ’ phase, the average radius of γ’ phase atoms was calculated according to Formula (1) [32]:

r_avgγ’_ = c_Alγ’_·r_Al_ + c_Crγ’_·r_Cr_ + c_Coγ’_·r_Co_ + c_Moγ’_·r_Mo_ + c_Tiγ’_· r_Ti_ + c_Taγ’_·r_Ta_ + c_Reγ’_·r_Re_ + c_Hfγ’_·r_Hf_ + c_Niγ’_·r_Ni_ (1)

where: r_avgγ’_—the average radius of the γ’ phase atoms; r_X_—average atom radius of element X: r_Al_ = 0.143 nm, r_Cr_ = 0.125 nm, r_Co_ = 0.125 nm, r_Mo_ = 0.136 nm, r_Ti_ = 0.145 nm, r_Ta_ = 0.149 nm, r_Re_ = 0.137 nm, r_Hf_ = 0.159 nm, r_Ni_ = 0.125 nm; c_Xγ’_—content of element X in γ‘ phase (% at.).

The lattice parameter of γ’ (aγ’) phase was calculated according to Formula (2):

aγ’ = (4·ravgγ’)/√2(2)

The parameter of long-range atomic order—S_d_—of the γ’ phase was determined by Mössbauer spectroscopy [33]. The value of this parameter depends on the specific location of the Al, Ti and Ta atoms in the lattice of the γ’ phase. The parameter S_d_ = 1, if the γ’ phase is completely ordered—Ni3Al(Ti,Ta). The value of Sd obtained from Mӧssbauer spectroscopy was applied to calculate probability P_(Al,Ti,Ta)_ of occupancy of the 000 atomic position in the crystal unit cell of the γ’ phase by Al, Ti, Ta using Equation (3) [34]:

P_(Al,Ti,Ta)_ = S_d_ (1-c_(Al,Ti,Ta)_) + c_(Al,Ti,Ta)_ (3)

where: P_(Al,Ti,Ta)_—probability of occupancy of the 000 atomic position by Al, Ti, Ta atoms; c_(Al,Ti,Ta)_—atomic concentration in γ’ phase; S_d_—parameter of long-range order of atomic arrangement in the γ’ phase.

Determination of the crystal orientation was conducted by an X-ray Ω-scan method using OD-EFG diffractometer (EFG, Berlin, Germany) [35]. The value of the deviation angle α_z_ between the direction of the mold withdrawal and [001] crystal directions was measured (Figure 1).

Creep resistance of the CMSX-4 superalloy solidified at various withdrawal rates was determined using the Walter + Bai AG LFMZ-30 electromechanical creep machine (Löhningen, Switzerland). The creep tests and sample preparation were carried out in accordance with the requirements of ASTM E139-11. Specimen holders made of Inconel 713C nickel superalloy with a columnar microstructure were used. Specimens were heated in the furnace to 982 °C in air and held at this temperature for 60 minutes. After this time, they were loaded with a constant axial force, causing an initial tensile stress of σ_r_ = 151.8 MPa. The temperature during the creep test was measured by 3 S-type PtRh10-Pt thermocouples.

## 3. Results and Discussion

**Figure 2:**
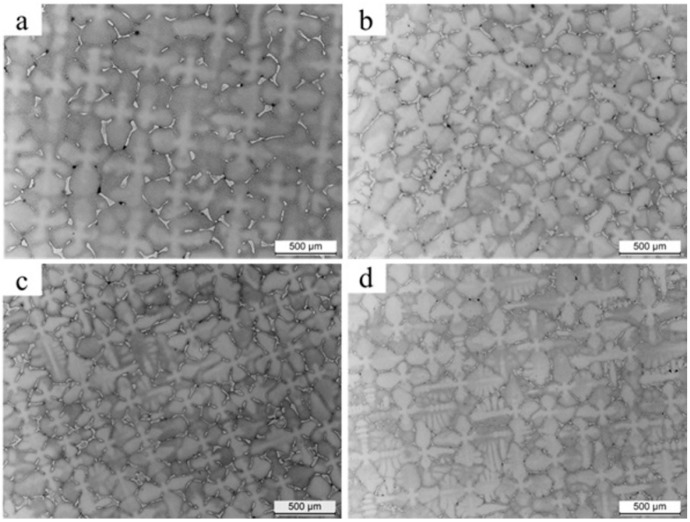
Dendritic microstructure (LM) of CMSX-4 superalloy solidified at different withdrawal rate: (**a**) 1, (**b**) 3, (**c**) 5 and (**d**) 7 mm/min.

LM analysis confirmed the dendritic microstructure in the castings manufactured at a withdrawal rate of 1, 3, 5 and 7 mm/min (Figure 2). The distance between the primary dendrite arm spacing (PDAS) was measured as follows (standard deviation): 457 (13), 376 (20), 321 (18) and 386 (15) μm for 1, 3, 5 and 7 mm/min, respectively. The increase in the withdrawal rate from 1 to 5 mm/min caused a decrease in PDAS. For 7 mm/min, PDAS was higher than expected but it should be noted that the standard deviation of the measured value was in the range of 13–20 μm. It was found, based on microstructure analysis (Figure 2), that for a higher withdrawal rate, more dendrites with tertiary arms grew. However, they were not observed in castings manufactured at 1 mm/min.

**Figure 3:**
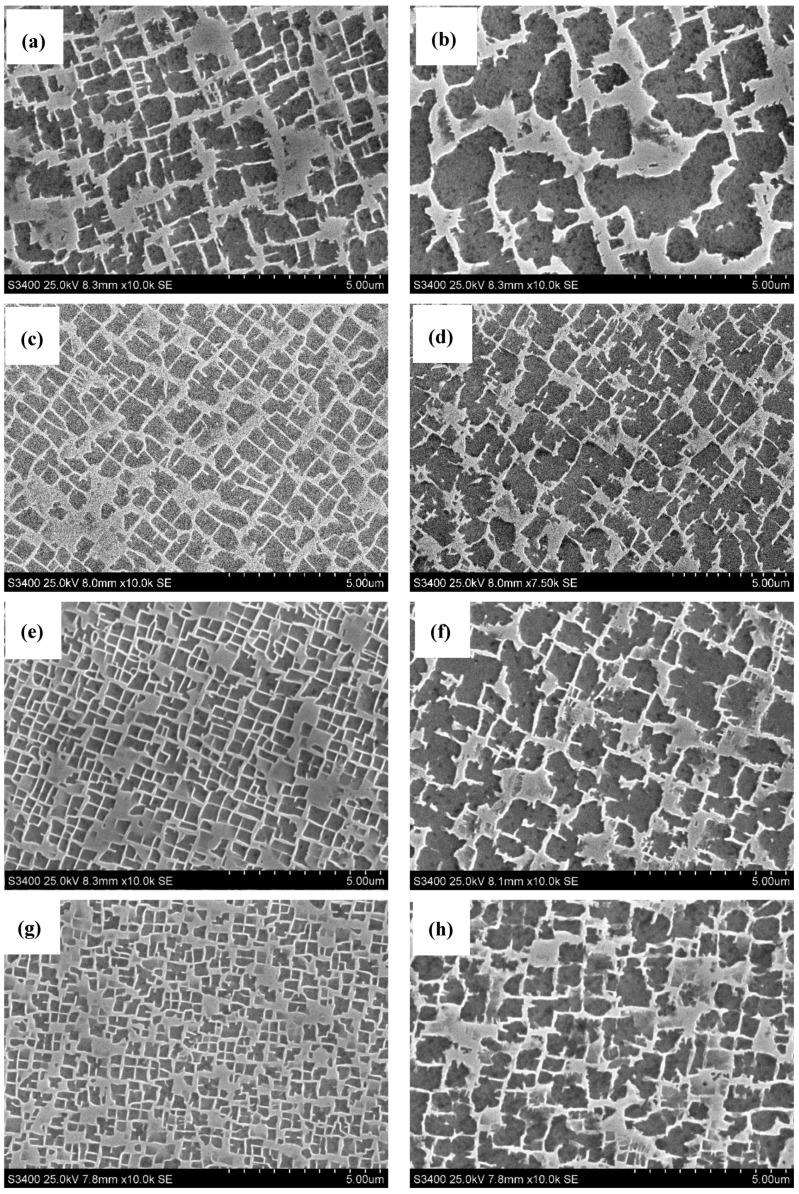
Morphology of γ’ precipitates in the matrix of γ phase in CMSX-4 superalloy solidified at different withdrawal rates: (**a**) 1, (**c**) 3, (**e**) 5 and (**g**) 7 mm/min—dendritic region, (**b**) 1, (**d**) 3, (**f**) 5 and (**h**) 7 mm/min—interdendritic region.

The SEM observation revealed the presence of the γ’ phase in microstructure of castings. In all cases, the γ’ precipitates were characterized by different, polyhedral morphology in dendritic and interdendritic regions. It was found that the increase in the withdrawal rate led to a decrease in the size of the γ’ precipitates (Figure 3).

**Figure 4:**
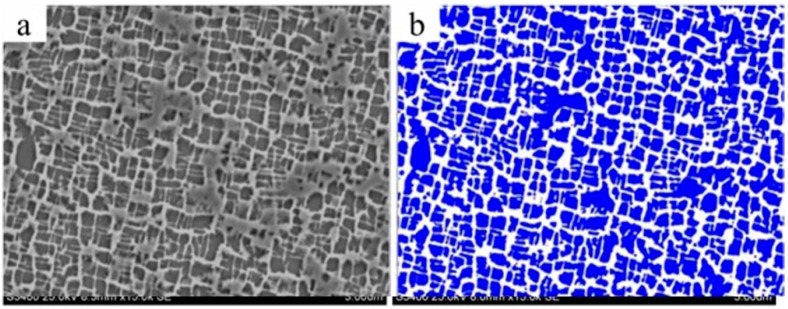
An example of image processing (binarization) to determine the volume fraction of microstructure constituents: (**a**) initial greyscale image (for microstructure of casting withdrawn at the rate of 1 mm/min), (**b**) binary image.

Stereological parameters of γ’ precipitates were determined using binary images (Figure 4). Castings withdrawn with the rate of 1 mm/min are characterized by average values of the γ’ precipitate surface area Sγ’ = 0.64 μm^2^ and the largest difference between its minimum and maximum values (2.03 μm^2^—dendritic; 6.12 µm^2^—interdendritic region) (Table 1). In the castings manufactured at a withdrawal rate of 3 mm/min, the Sγ’ = 0.31 μm^2^—half of the value obtained at 1 mm/min. The smallest scatter of Sγ’ values (0.35 μm^2^, Table 1) was found in the microstructure of castings withdrawn at the rate of 5 mm/min in the dendritic region. The size of γ’ precipitates decreased significantly with an increase in the withdrawal rate from 1 to 5 mm/min; the difference between data calculated for 5 and 7 mm/min is much smaller (Table 1). The sizes of precipitations of γ’ are bigger in the interdendritic region then in dendrite one for 1, 3, 5 and 7 mm/min castings.

The highest volume fraction of the γ’ phase precipitations was found in the casting withdrawn at the rate of 5 mm/min—V_Vγ’_ = 64.6% (58.7—dendritic; 68.7—interdendritic region) (Table 2). Then, for casting at a withdrawal rate 1 mm/min and the smallest volume fractions were found for 3 and 7 mm/min. A small difference in the V_Vγ’_ average values—about 5%—and high values of standard deviation for 7 mm/min (11.1) make it not possible to evaluate the effect of the withdrawal rate on the volume fraction of γ’ precipitations (Table 2). The volume fraction of γ’ is bigger in the interdendritic region than the dendritic in castings at withdrawal rates of 1, 3, 5 and 7 mm/min. Additionally, the accuracy of such measurements strongly depend on subjective assessment in the image binarization process (Figure 4).

The chemical composition of the γ’ phase was determined by the EDS method. Among alloying elements of CMSX-4 superalloy, those which form the γ’ phase were separately analysed—Ni, Al, Ti and Ta (Table 3). The total content of Al, Ti and Ta in the γ’ phase decreased with an increase in the withdrawal rate from 1 to 5 mm/min (Table 3). The content of Ni in the γ’ phase was the highest at a withdrawal rate of 3 mm/min—76.23% at. Then, for withdrawal rates of 1 and 7 mm/min, the value of nickel is similar—around 75%. The lowest amount of nickel was found in the casting made at the withdrawal rate of 5 mm/min.

The calculated values of the lattice parameter of the phases indicate that the a_γ’_ decreases with the increase in the withdrawal rate. The values of the a_γ_ were the highest for the withdrawal rate of 3 mm/min. It was then reduced for withdrawal rates of 7, 5 and 1 mm/min. The largest difference between the a_γ_ and a_γ’_ values was found for a withdrawal rate of 1 mm/min, the smallest was observed for 7 mm/min (Table 4).

**Figure 5:**
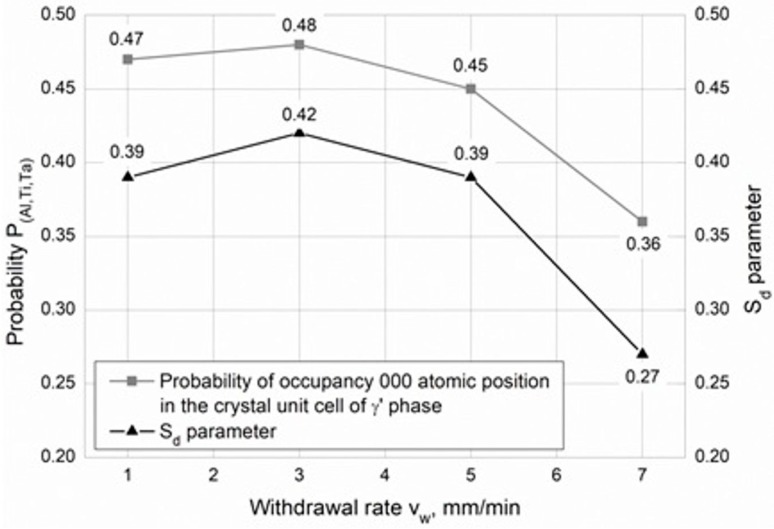
Values of probability of occupancy of the 000 atomic position by Al, Ti and Ta and S_d_ parameters as a function of the withdrawal rate.

On the basis of the Mössbauer spectroscopy analysis, it was found that the values of the S_d_ parameter (the long-range order of atomic arrangement) are similar for the withdrawal rates 1–5 mm/min—about 0.4 (Figure 5). A significantly lower value was determined for casting withdrawn at the rate of 7 mm/min. The probability of occupancy of the 000 atomic position shows that the most ordered γ’ phase is present in castings withdrawn at the rate of 1–5 mm/min—from 0.45 to 0.48 (Figure 5). Less order was observed for casting crystalized at the withdrawal rate 7 mm/min—0.36 (Figure 5). It seems that the ordering of the γ’ phase decreases when the withdrawal rate in the Bridgman process is greater than 3 mm/min.

The obtained results showed that the γ’ phase in the CMSX-4 nickel-based superalloy is not completely ordered. It consists of a part of the ordered phase with the Pm3m space group and the disordered solid solution with the Fm3m space group.

**Figure 6:**
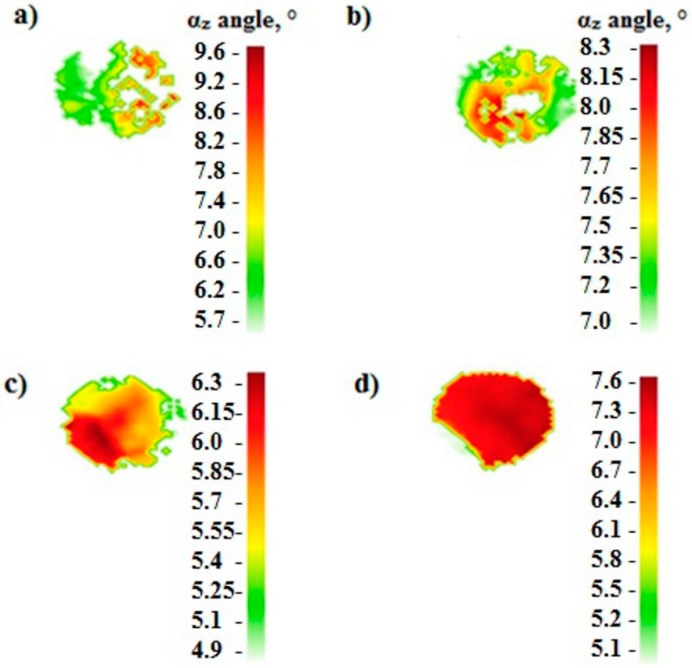
The values of the αz angle on the surface of cross-section of the CMSX-4 superalloy casting withdrawn at the rate of: (**a**) 1, (**b**) 3, (**c**) 5, (**d**) 7 mm/min.

The crystal orientation determined by the Ω-scan method on the cross-sections of castings showed that the α_z_ angle for all four investigated withdrawal rates takes values well below the acceptable maximum of 15° (Table 5). Castings differ between the minimum and maximum values of the α_z_ angle measured on the casting cross-sections. For the casting made at a withdrawal rate of 1 mm/min, the α_z_ angle takes the values in the range from 5.7° to 9.6° (Figure 6a). Hence, the scatter of this angle on the analyzed surface was 3.9°. For the casting produced at 3 mm/min, the α_z_ angle takes the value from 7° to 8.3°—the difference was 1.3° (Figure 6b). A similar difference between the minimum and the maxim α_z_ was for a withdrawal rate of 5 mm/min, where the α_z_ angle min = 4.9 and max = 6.2° (Figure 6c). For a withdrawal rate of 7 mm/min, α_z_ ranges from 5.1° to 7.6° (Figure 6d). In summary, the withdrawal rate did not affect the value of the αz angle in single crystal castings.

**Figure 7:**
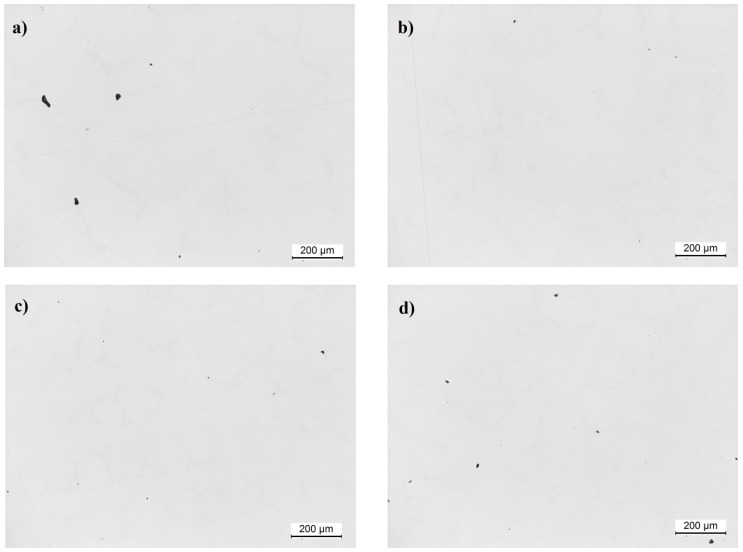
Micrographs of pores of cross-section of the CMSX-4 superalloy casting withdrawn at the rate of: (**a**) 1, (**b**) 3, (**c**) 5, (**d**) 7 mm/min.

**Figure 8:**
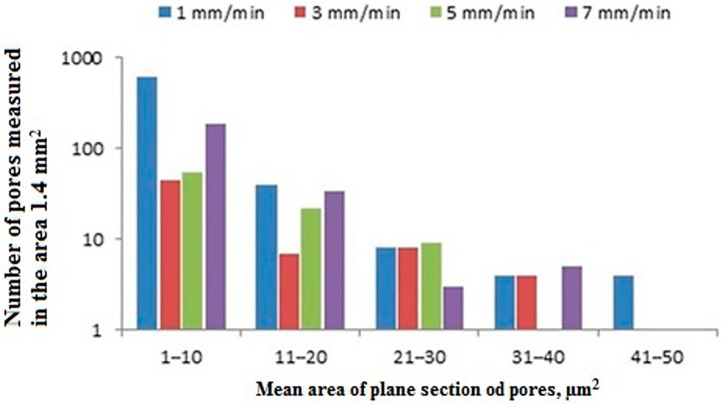
The number of pores and their average area measured in the cross-section of the single crystal (SX) castings.

**Figure 9:**
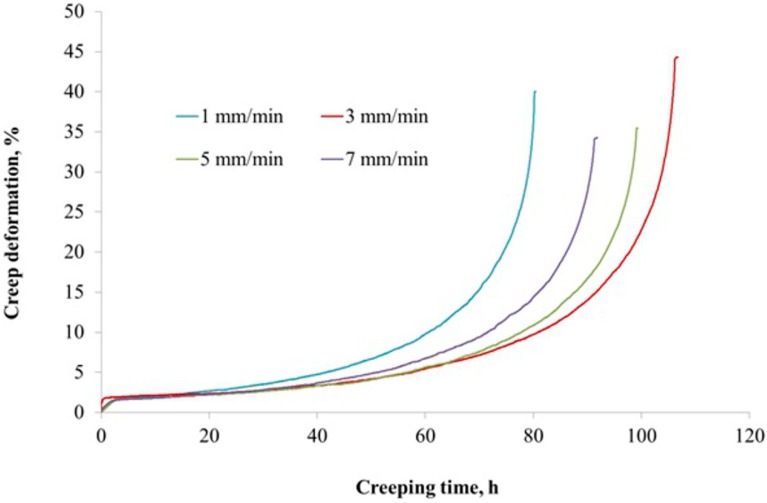
Creep curves of single crystal castings.

Analysis of the porosity (Figure 7) showed significant differences in the number on the surface of pores in 1.4 mm^2^ area and their size (Figure 8). The largest number of pores, with a surface area of up to 10 μm^2^, was found in a casting made at the withdrawal rate of 1 mm/min. Castings produced at the withdrawal rate of 7 mm/min also contained a large number of pores; however, fewer than in the 1 mm/min casting. The lowest number of pores was found in the castings withdrawn at the rate of 3 and 5 mm/min. The biggest pores (41–50 µm^2^)— the larger the pores the greater the reduction in the mechanical properties—were detected in castings withdrawn at rate of 1 mm/min only (Figure 9).

Creep tests showed differences between the castings both in terms of time to failure and total creep deformation. The longest creep life was found for the casting solidified at a withdrawal rate of 3 mm/min—111 h. The measured creep deformation was 44%. The casting with a withdrawal rate at 5 mm/min was broken after a test duration of 102 h and the measured creep deformation was 35%. For the 7 mm/min casting, the creep time was 94 h and the creep deformation, approximated as for 5 mm/min, was 34%. The shortest time for sample destruction to occur (83 h) was found in the sample for casting 1 mm/min and the creep deformation was equal to 40% (Figure 9). Creep life data obtained for various castings coincided with the results of the porosity analysis. The casting produced at the withdrawal rate of 1 mm/min that showed the largest number of pores on its surface, exhibited the shortest time to failure in the creep test. The casting produced at the withdrawal rate of 3 mm/min, which showed the lowest number of pores on its surface, was characterized by the longest creep time. Hence, it can be concluded that its porosity had the greatest influence on the creep strength of the casting.

The microstructure of a CMSX-4 superalloy depends on the conditions of solidification, which are determined by the casting production process. In the Bridgman method, the temperature gradient and cooling rate are much lower than in the LMC method. An increase of these solidification parameters leads to a refinement of the microstructure and a reduction of the primary dendrites arm spacing (PDAS). PDAS determines the size of the pores that form under the solidification of the mushy zone. It is well known that the shape and size of the pores have a decisive impact on the mechanical properties of nickel-based superalloy castings. The high-cycle fatigue life of castings increases significantly for PDAS = 250 μm, which is refined by the LMC method [36,37,38]. In the presented study, the highest creep resistance was detected for castings withdrawn with the rate of 3 and 5 mm/min (Figure 9), characterized by the smallest PDAS values (376 and 321 μm respectively) and porosity (Figure 8).

## 4. Conclusions

The crystal structure perfection of the CMSX-4 superalloy was evaluated for castings manufactured at the withdrawal rate of 1, 3, 5 and 7 mm/min using microscopic, X-ray Ω-scan and Mössbauer spectroscopy methods. The effect of the level of the crystal structure perfection on creep behavior was also analyzed. Based on the obtained results, the following conclusions can be formulated:

1. Porosity has the greatest impact on the creep resistance of single crystal castings. Increased pore content leads to reduced creep resistance. Castings containing a large number of pores are broken after a shorter time.
2. The withdrawal rate of castings in the employed range (1–7 mm/min) does not affect the crystal orientation. For each adopted withdrawal rate, the casting was obtained in the [001] crystal orientation (α_z_ < 15°).
3. There was no influence of withdrawal rate on the volume fraction of the γ’ phase. The volume fraction of this phase in “as-cast” CMSX-4 superalloy is close to approximately 60%.
4. The value of the lattice parameter a_0γ’_ decreases with the increase in the withdrawal rate.

## Figures and Tables

**Table 1:** The chemical composition of the CMSX-4 single crystal superalloy.

Element Content, % wt.									
Cr	Co	Mo	W	Ta	Al	Ti	Hf	Re	Ni
6.5	9	0.6	6	6.5	5.6	1	0.1	3	bal.

**Table 2:** Volume fraction and surface area of γ’ phase precipitates in CMSX-4 superalloy in the as-cast state, depending on the rate of single crystal withdrawal.

Withdrawal Rate v_w_, mm/min	Relative Volume of γ’ Phases V_Vγ’_, %			Surface Area of γ’ Phase S_γ’_, μm^2^					
	Average	Dendritic	Interdendritic Region	Dendritic			Interdendritic Region		
				Min	Max	Ave	Min	Max	Ave
1	61.1 (0.6)	55.1	69.2	0.05	2.08	0.64	0.08	6.2	1.13
3	59.9 (2.0)	54.8	65.8	0.09	1.0	0.31	0.09	4.1	0.55
5	64.6 (3.7)	58.7	68.7	0.07	0.35	0.16	0.05	3.7	0.53
7	59.9 (11.1)	60.1	65.2	0.02	0.38	0.09	0.05	3.4	0.46

**Table 3:** Chemical composition of the γ’ phase in CMSX-4 superalloy.

		Withdrawal Rate vw, mm/min			
		1	3	5	7
Elements content in γ’ phase, % at. (standard deviation)	Cr	2.5 (0.5)	2.3 (0.7)	5.4 (2.8)	3.2 (1.1)
	Co	7.3 (0.4)	8.2 (0.5)	10.09 (2.3)	8.2 (0.8)
	Mo	0.3 (0.2)	0.3 (0.1)	0.5 (0.2)	0,1 (0.1)
	W	0.9 (0.1)	1.1 (0.1)	3.5 (1.0)	2,2 (0.4)
	Ta	1.7 (0.2)	1.5 (0.3)	4.0 (1.3)	5.4(0.5)
	Re	0.3(0.1)	0.2 (0.1)	1.9(0.9)	0.4 (0.1)
	Al	10.6 (0.5)	8.5 (1.4)	3.7 (0.9)	4.5(0.3)
	Ti	1.1 (0.2)	1.1 (0.15)	0.7 (0.3)	1.1 (0.2)
	Ni	75.3 (1.0)	76.2 (1.4)	70.2 (4.9)	75.1 (0.6)
	Al+Ta+Ti	13.4	11.1	10.4	12.7

**Table 4:** Lattice parameter of the γ and γ’ phases.

Withdrawal Rate vw, mm/min	Lattice Parameter a, nm (Standard Deviation)	
	a_γ_	a_γ’_
1	0.3563 (0.0005)	0.3604 (0.0006)
3	0.3571 (0.0012)	0.3604 (0.0008)
5	0.3568 (0.0004)	0.3594 (0.0011)
7	0.3564 (0.0008)	0.3589 (0.0008)

**Table 5:** Value of αz angle [°] between the [001] crystal direction and the direction of mold withdrawal.

Withdrawal Rate vw, mm/min											
1			3			5			7		
α_z_ min	α_z_ max	Δ α_z_	α_z_ min	α_z_ max	Δ α_z_	α_z_ min	α_z_ max	Δ α_z_	α_z_ min	α_z_ max	Δ α_z_
5.7	9.6	3.9	7.0	8.3	1.3	4.9	6.3	1.4	5.1	7.6	2.5
